# Supplementary material for: Low 13C-13C abundances in abiotic ethane
Source: Nat Commun. 2022 Oct 2;13:5790. doi: 10.1038/s41467-022-33538-9 (PMC9527245; doi:10.1038/s41467-022-33538-9)
Supplement: Supplementary file 1 — Supplementary Information [file 41467_2022_33538_MOESM1_ESM.pdf]

Supplementary Information:

Low ^{13}C-^{13}C abundances in abiotic ethane

Koudai Taguchi, Alexis Gilbert, Barbara Sherwood Lollar, Thomas Giunta, Christopher J. Boreham, Qi Liu, Juske Horita, & Yuichiro Ueno

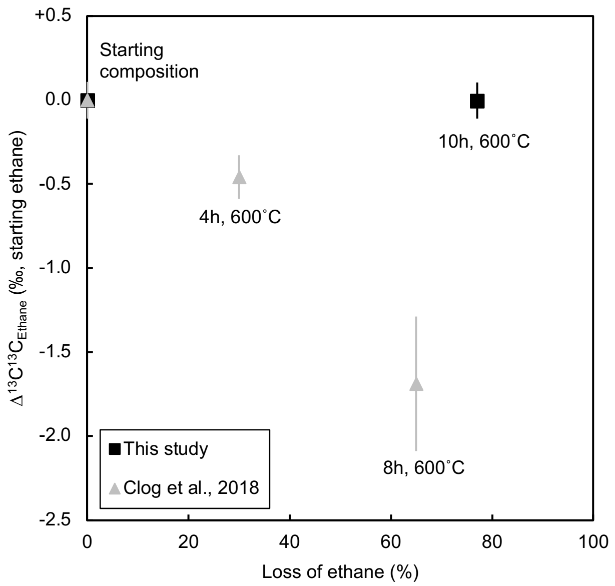

Supplementary Fig. 1| Results of $\Delta^{13}C^{13}C$ from ethane pyrolysis experiment at 600°C. Relationship between $\Delta^{13}C^{13}C$ and amount of ethane costed by heating at 600°C. The $\Delta^{13}C^{13}C$ values was represented as a deviation from starting ethane.

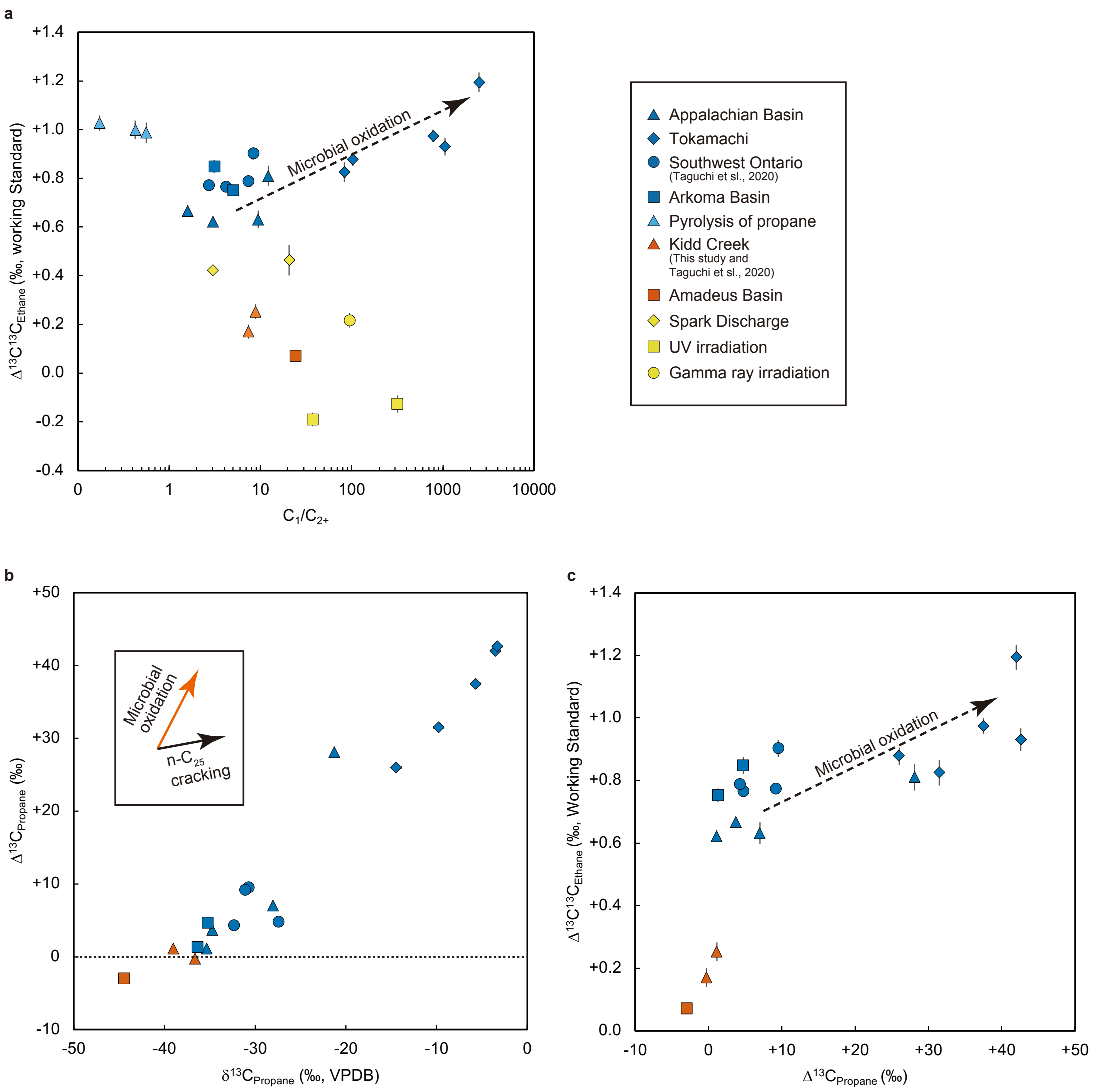

Supplementary Fig. 2| $\Delta^{13}C^{13}C$ indicates microbial oxidation of ethane. The dotted arrows show the predicted direction of change due to microbial oxidation of ethane in $\Delta^{13}C^{13}C$. **a,** $\Delta^{13}C^{13}C$ as a function of C_1/C_{2+} ratios. **b,** bulk isotope composition ($\delta^{13}C_{Propane}$) as a function of $\Delta^{13}C_{Propane}$. The plotted trend is based on the calculation from experiments[1]. Biodegradation trend from incubation experiments of propane is represented by red symbols; thermogenic trend from cracking experiments of an n-C_{25} alkane is represented by black

symbols[1]. **c,** $\Delta^{13}C^{13}C$ vs. position-specific carbon isotope composition of propane (= $\Delta^{13}C_{Propane}$). $\Delta^{13}C_{Propane}$ represents the difference of $^{13}C/^{12}C$ ratio between the central and terminal carbon position of propane.

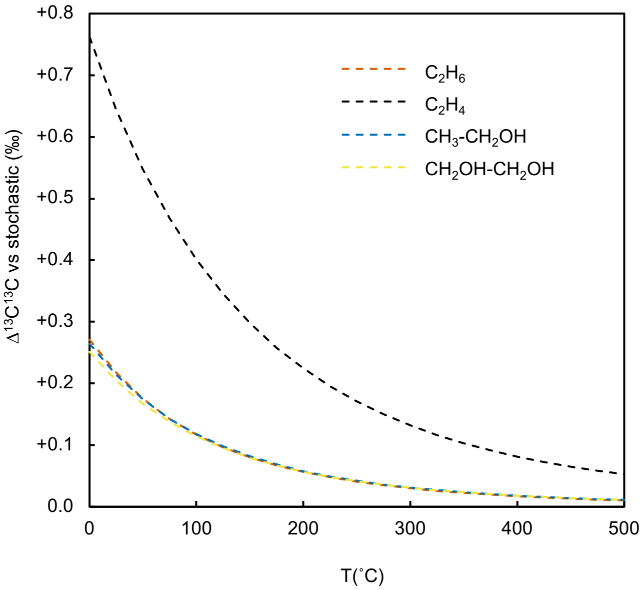

Supplementary Fig. 3| $\Delta^{13}C^{13}C$ relative to stochastic distribution as a function of temperature for a given molecule. The $\Delta^{13}C^{13}C$ values are calculated from equilibrium constants from eq. (9) and eq. (10) (see Methods).

Supplementary Table 1| Sample location, sample ID, carbon isotopes, and clumped-isotope data of ethane.

Geological Setting /Experiments	Sample ID	$\delta^{13}C_{C2H6}$	$\Delta^{13}C^{13}C$ vs W.S.	SE	$\Delta^{13}C^{13}C$ vs stochastic	SE
Appalachian Basin	Amherst	-33.6	0.63	0.04	-0.10	0.05
	Barcelona Springs	-43.2	0.62	0.02	-0.11	0.03
	Gasport	-34.3	0.81	0.04	0.08	0.06
	Eternal Fame	-39.9	0.67	0.01	-0.06	0.03
Southwest Ontario Basin	T008638B	-28.3	0.77	0.01	0.04	0.03
	T002235	-37.0	0.90	0.03	0.17	0.04
	T004918	-38.2	0.77	0.01	0.04	0.02
	T009877	-37.2	0.79	0.01	0.06	0.03
Arkoma Basin	Eddings 3H20	-40.9	0.85	0.03	0.12	0.04
	Martin 1H35	-42.1	0.75	0.02	0.02	0.04
Tokamachi	T19-M18	-26.3	0.88	0.03	0.15	0.04
	T19-G11	-21.2	0.83	0.04	0.10	0.06
	T21-M35	-22.0	0.97	0.02	0.24	0.04
	T21-M22	-21.0	1.19	0.04	0.46	0.05
	T21-MA4	-22.5	0.93	0.04	0.20	0.05
Kidd Creek Mine	KCL7850BH12299	-39.8	0.25	0.03	-0.48	0.04
	KCL9500BH13684	-37.0	0.17	0.03	-0.56	0.04
Amadeus Basin	Amadeus Dingo 3	-42.9	0.07	0.02	-0.66	0.03
UV irradiation	UV-3h	-59.1	-0.13	0.04	-0.86	0.05
	UV-16h	-58.9	-0.19	0.03	-0.92	0.04
Gamma-Ray Irradiation	19 CH4	-51.4	0.22	0.03	-0.51	0.04
Spark Discharge	Spark-15min	-48.8	0.46	0.06	-0.27	0.08
	Spark-5h	-35.3	0.42	0.01	-0.31	0.03
Pyrolysi of propane	PyC3-15h	-33.4	1.03	0.03	0.30	0.05
	PyC3-30h	-32.2	1.00	0.04	0.27	0.05
	PyC3-45h	-29.1	0.99	0.04	0.26	0.06

W.S. = Working Standard

Supplementary Table 2| Sample location, sample ID, concentration, and carbon isotopes.

Geological Settings	Sample ID	Concentration (%) CH_4	CO_2	C_2H_6	C_3H_8	i-C_4H_{10}	n-C_4H_{10}	$\delta^{13}C$ (‰) CH_4	CO_2	C_2H_6	C_3H_8	i-C_4H_{10}	n-C_4H_{10}
Appalachian	Amherst	90.3	0.1	7.6	1.6	0.20	0.19	-42.1	-18.7	-33.6	-28.0	-28.7	-27.6
Basin	Barcelona Springs	74.9	0.3	15.7	7.5	0.36	1.28	-51.5	13.1	-43.2	-35.3	-33.9	-33.1
	Gasport	95.4	0.6	7.5	0.3	0.01	0.0016	-43.7	35.7	-34.3	-21.3	-23.4	-15.6
	Eternal Fame	60.9	0.9	23.2	11.6	0.90	2.40	-49.3	11.5	-39.9	-34.7	-33.3	-32.0
Southwest	T008638-B	76.6	-	10.9	4.8	1.10	1.40	-39.5	-	-28.3	-27.4	-26.1	-25.9
Ontario	T002235	81.1	-	5.7	2.6	0.70	0.70	-51.3	-	-37.0	-30.7	-28.9	-28.8
	T004918	68.8	-	11.5	8.0	3.10	2.80	-51.8	-	-38.2	-31.1	-30.2	-29.1
	T009877	78.5	-	7.2	2.6	0.30	0.50	-46.0	-	-37.2	-32.3	-30.3	-29.2
Arkoma	Eddings 3H20	74.4	-	15.1	6.5	0.70	1.60	-51.3	-	-40.9	-35.2	-33.6	-32.8
Basin	Martin 1H35	81.9	-	8.6	5.1	0.70	1.90	-42.4	-	-42.1	-36.3	-34.2	-34.1
Tokamachi	T19-M18	94.4	4.7	0.59	0.32	n.a.	n.a.	-36.8	34.1	-26.3	-14.4	n.a.	n.a.
	T19-G11	96.7	2.2	0.74	0.10	0.26	0.05	-34.1	28.5	-21.2	-9.8	-24.3	-14.6
	T21-M35	96.1	3.8	0.08	0.04	0.0007	0.0005	-35.3	33.4	-22.0	-5.7	n.a.	n.a.
	T21-M22	94.8	5.1	0.03	0.01	0.0005	0.0004	-35.1	34.0	-21.0	-3.5	n.a.	n.a.
	T21-MA4	95.3	4.6	0.08	0.01	0.001	0.0004	-34.8	34.4	-22.5	-3.3	n.a.	n.a.
Kidd Creek	KCL7850 BH12299	74.6	-	7.23	0.95	0.1	0.2	-39.7	-	-39.8	-39.0	-42.8	-38.1
	KCL9500 BH13684	75.2	-	8.30	1.50	0.1	0.3	-31.9	-	-37.0	-36.6	-39.1	-36.0
Amadeus	Amadeus Dingo 3	96.0	-	3.47	0.38	0.1	0.1	-34.9	-	-42.9	-44.4	-47.2	-46.8

n.a. = not analysed

Supplementary Table 3| Carbon isotopes and clumped-isotope data of ethane from pyrolysis experiment of ethane at 600˚C.

Sample	$\delta^{13}C$	SE	$\Delta^{13}C^{13}C$ vs. W.S.	SE	Ethane yield
Start	21.983	0.004	0.90	0.11	100%
10h 600˚C	24.180	0.004	0.89	0.06	77%

W.S. = Working Standard

Supplementary Table 4| Sample location, sample ID, carbon isotopes, and intramolecular ^{13}C distribution in propane.

Geological Settings	Sample ID	$\delta^{13}C$ (‰)	$\Delta^{13}C_{propane}$
Appalachian Basin	Amherst	-28.0	7.0
	Barcelona Springs	-35.3	1.1
	Gasport	-21.3	28.1
	Eternal Fame	-34.7	3.7
Southwest Ontario	T008638-B	-27.4	4.8
	T002235	-30.7	9.5
	T004918	-31.1	9.2
	T009877	-32.3	4.3
Arkoma Basin	Eddings 3H20	-35.2	4.7
	Martin 1H35	-36.3	1.3
Tokamachi	T19-M18	-14.4	26.0
	T19-G11	-9.8	31.5
	T21-M35	-5.7	37.5
	T21-M22	-3.5	42.0
	T21-MA4	-3.3	42.6
Kidd Creek Mine	KCL7850BH12299	-39.0	1.1
	KCL9500BH13684	-36.6	-0.3
Amadeus Basin	Dingo 3	-44.4	-3.0

Supplementary Table 5| $\Delta^{13}C^{13}C$ compared to the stochastic distribution under thermodynamic equilibrium at each temperature.

	$\Delta^{13}C^{13}C$ vs. Stochastic (‰)			
Temp. (°C)	C_2H_6	C_2H_4	CH_3-CH_2OH	CH_2OH-CH_2OH
0	0.27	0.76	0.26	0.25
25	0.22	0.64	0.21	0.20
50	0.18	0.55	0.17	0.17
100	0.12	0.40	0.12	0.11
200	0.06	0.22	0.06	0.06
300	0.03	0.13	0.03	0.03
400	0.02	0.08	0.02	0.02
500	0.01	0.05	0.01	0.01

Supplementary Table 6| Experiments, sample ID, concentration, carbon isotopes of hydrocarbons.

		Concentration (%)						
Experiments	Sample ID	CH_4	C_2H_4	C_2H_6	C_3H_8	i-C_4H_{10}	n-C_4H_{10}	
UV irradiation	UV-3h	98.8	n.a.	0.3	0.04	0.002	0.002	
	UV-16h	96.6	n.a.	2.4	0.16	0.006	0.007	
Gamma-ray irradiation	19 CH4	99.0	n.a.	0.88	0.09	0.02	0.04	
Spark Discharge	Spark-15min	95.1	0.3	3.2	1.0	0.2	0.2	
	Spark-5h	74.9	0.3	16.0	6.2	1.2	1.4	
		$\delta^{13}C$ (‰)						Starting
Experiments	Sample ID	CH_4	C_2H_4	C_2H_6	C_3H_8	i-C_4H_{10}	n-C_4H_{10}	CH_4
UV irradiation	UV-3h	-42.9	n.a.	-59.1	-64.0	-63.4	-64.8	n.a.
	UV-16h	-47.0	n.a.	-58.9	-60.6	-60.5	-63.0	-48.3
Gamma-ray irradiation	19 CH4	-43.8	n.a.	-51.4	-49.3	-49.2	-50.1	-45.7
Spark Discharge	Spark-15min	-42.3	n.a.	-48.8	-46.9	-46.5	-45.5	-42.4
	Spark-5h	-31.2	-33.1	-35.3	-36.2	-35.2	-36.1	-51.8

n.a. = not analysed; n.d. = not detected

Supplementary Table 7| Experiments, sample ID, concentration, carbon isotopes of hydrocarbons.

		Concentration (%)					$\delta^{13}C$ (‰)					Starting
Experiment	Sample ID	CH_4	C_2H_4	C_2H_6	C_3H_6	C_3H_8	CH_4	C_2H_4	C_2H_6	C_3H_6	C_3H_8	C_3H_8
Pyrolysis	Pyr. C3H8-15h	14.7	8.8	3.6	7.5	65.4	-32.8	-33.4	-37.9	-29.6	-29.4	-29.2
of propane	Pyr. C3H8-30h	29.8	10.2	10.6	8.3	41.2	-35.2	-32.2	-38.7	-28.4	-26.3	-29.2
	Pyr. C3H8-45h	35.8	9.0	13.6	7.6	34.0	-33.7	-29.1	-36.0	-25.7	-27.5	-29.2

Supplementary Note 1| Kinetic isotope effect in abiotic ethane

Kinetic isotope effect may also play a role in ^{13}C-^{13}C signature from abiotic samples associated with abiotic polymerization step. To estimate the kinetic isotope effect, three simplified polymerization reaction were considered as follows:

$$^{12}CH_3 + {}^{12}CH_3 \rightarrow {}^{12}CH_3{}^{12}CH_3 \qquad (1)$$

$$^{12}CH_3 + {}^{13}CH_3 \rightarrow {}^{12}CH_3{}^{13}CH_3 \qquad (2)$$

$$^{13}CH_3 + {}^{13}CH_3 \rightarrow {}^{13}CH_3{}^{13}CH_3 \qquad (3)$$

Reaction rate for each polymerization reactions can be defined using reaction rate constants k, k', and k'' as follows:

$$d[^{12}CH_3{}^{12}CH_3]/dt = k[^{12}CH_3][^{12}CH_3] \qquad (4)$$

$$d[^{12}CH_3{}^{13}CH_3]/dt = 2 \times k'[^{12}CH_3][^{13}CH_3] \qquad (5)$$

$$d[^{13}CH_3{}^{13}CH_3]/dt = k''[^{13}CH_3][^{13}CH_3] \qquad (6)$$

Then, $^{13}R_{Ethane}$ and $^{1313}R_{Ethane}$ can be obtained by dividing eq.5 by eq.4 and 2 and eq.6 by eq.4 considering the symmetry of two carbon atoms in ethane as follows:

$$^{13}R_{Ethane} = (k'/k) \times {}^{13}R_{Methyl} \qquad (7)$$

$$^{1313}R_{Ethane} = (k''/k) \times {}^{13}R_{Methyl}{}^{2} \qquad (8)$$

where $^{13}R_{Methyl}$ represents $^{13}C/^{12}C$ ratio calculated from $[^{13}CH_3]/[^{12}CH_3]$. Consequently, the

$\Delta^{13}C^{13}C$ value can be calculated as follow:

$$\Delta^{13}C^{13}C = \ln[(k''/k)/(k'/k)^2] \qquad (9)$$

Therefore, the $\Delta^{13}C^{13}C$ value associated with kinetic isotope effect through polymerization reaction depends on the coefficient $(k''/k)/(k'/k)^2$, leading to depleted ^{13}C-^{13}C signature $[(k''/k) < (k'/k)^2]$ or enriched ^{13}C-^{13}C signature $[(k''/k) > (k'/k)^2]$ relative to stochastic distribution. If $\Delta^{13}C^{13}C$ value has stochastic distribution, (k''/k) is equal to $(k'/k)^2$.

The recombination reaction between CH_3 radical is via the formation of C_2H_6* with high energy (10) and deactivation to C_2H_6 by its collision with another molecule/atom (third body) M (11) as follow:

$$CH_3 + CH_3 \rightleftarrows C_2H_6^* \qquad (10)$$

$$C_2H_6^* + M \rightarrow C_2H_6 + M \quad (11)$$

The pressure of our experiments (about 12kPa of CH_4) is range of high-pressure-limit, leading to an almost quantitative conversion of C_2H_6* to C_2H_6. Therefore, the third body, M, does not contribute to the isotope effect during the experiment here.

Supplementary reference

1. Gilbert, A. et al. Intramolecular isotopic evidence for bacterial oxidation of propane in subsurface natural gas reservoirs. *Proc. Natl Acad. Sci. U. S. A.* **116**, 6653–6658 (2019).
